# Slow-wave sleep is controlled by a subset of nucleus accumbens core neurons in mice

**DOI:** 10.1038/s41467-017-00781-4

**Published:** 2017-09-29

**Authors:** Yo Oishi, Qi Xu, Lu Wang, Bin-Jia Zhang, Koji Takahashi, Yohko Takata, Yan-Jia Luo, Yoan Cherasse, Serge N. Schiffmann, Alban de Kerchove d’Exaerde, Yoshihiro Urade, Wei-Min Qu, Zhi-Li Huang, Michael Lazarus

**Affiliations:** 1 International Institute for Integrative Sleep Medicine (WPI-IIIS), University of Tsukuba, 1-1-1 Tennodai, Tsukuba, Ibaraki 305-8575 Japan; 2 Department of Pharmacology, State Key Laboratory of Medical Neurobiology, Institutes of Brain Science and the Collaborative Innovation Center for Brain Science, Shanghai Medical College of Fudan University, Shanghai, 200032 China; 3 Department of Physiology, School of Basic Medical Sciences, Anhui Medical University, Hefei, 230032 China; 4 Laboratory of Neurophysiology, ULB-Neuroscience Institute, Université Libre de Bruxelles (ULB), 808 Route de Lennik, Brussels, 1070 Belgium

## Abstract

Sleep control is ascribed to a two-process model, a widely accepted concept that posits homoeostatic drive and a circadian process as the major sleep-regulating factors. Cognitive and emotional factors also influence sleep–wake behaviour; however, the precise circuit mechanisms underlying their effects on sleep control are unknown. Previous studies suggest that adenosine has a role affecting behavioural arousal in the nucleus accumbens (NAc), a brain area critical for reinforcement and reward. Here, we show that chemogenetic or optogenetic activation of excitatory adenosine A_2A_ receptor-expressing indirect pathway neurons in the core region of the NAc strongly induces slow-wave sleep. Chemogenetic inhibition of the NAc indirect pathway neurons prevents the sleep induction, but does not affect the homoeostatic sleep rebound. In addition, motivational stimuli inhibit the activity of ventral pallidum-projecting NAc indirect pathway neurons and suppress sleep. Our findings reveal a prominent contribution of this indirect pathway to sleep control associated with motivation.

## Abstract (web summary)

In addition to circadian and homoeostatic drives, motivational levels influence sleep−wake cycles. Here the authors demonstrate that adenosine receptor-expressing neurons in the nucleus accumbens core that project to the ventral pallidum are inhibited by motivational stimuli and are causally involved in the control of slow-wave sleep.

## Introduction

Virtually all living organisms with a nervous system, ranging from worms to humans, exhibit sleep or sleep-like behaviour as a neurological function^1, 2^. Sleep regulation is conceptualised by the popular “two-process” model that posits homoeostatic and circadian drives control sleep^3^. The homoeostatic process is controlled by sleep pressure, which accumulates during the course of wakefulness and dissipates during sleep. In addition, the sleep–wake cycle during the day and night is regulated by a circadian process that is independent of prior sleep or wake time and is controlled by an internal biological clock located in the suprachiasmatic nucleus deep within the brain. Sleep/wake behaviour, however, is also influenced by cognitive and emotional factors^4–6^. Humans often defy sleepiness and stay awake when attention is necessary, but also experience an inescapable desire to sleep during boring situations. The mechanisms by which motivational stimuli interact with sleep/wake behaviour are not accounted for by the two-process model and the brain mechanisms governing the regulation of sleep by cognitive and emotional factors are largely unknown.

Neural systems that promote sleep have been identified in the brainstem and basal forebrain^7, 8^. Animals with lesions of these brain areas, however, still sleep a significant amount^9–11^, suggesting the existence of other sleep-inducing circuitry. Pharmacological studies^12–14^ suggest that the classic endogenous somnogen adenosine^15, 16^ affects behavioural arousal via excitatory adenosine A_2A_ receptors (A_2A_R) on neurons in the nucleus accumbens (NAc), a part of the brain that is associated with motivation, pleasure, and positive reinforcement^17, 18^. The mechanism by which these neurons play a key role in the control of sleep, however, is unknown.

In the present study, we demonstrate that selective activation of A_2A_R-expressing NAc neurons strongly induced sleep behaviour in mice, whereas inhibition of these neurons suppressed sleep. Based on optogenetic and anterograde-tracing experiments, we found that the core subdivision of the NAc and the ventral pallidum (VP) in the basal forebrain are key areas involved in generating sleep. Homoeostatic sleep pressure produced by sleep deprivation did not change the neuronal activity of NAc core A_2A_R neurons projecting to the VP based on immunohistochemical analysis of c-Fos expression. By contrast, typical motivational stimuli attenuated the activity of these neurons and reduced sleep amount. These data reveal the sleep-inducing role of NAc that is regulated by motivational factors.

## Results

### Chemogenetic activation of NAc A_2A_R neurons increases sleep

**Fig. 1:**
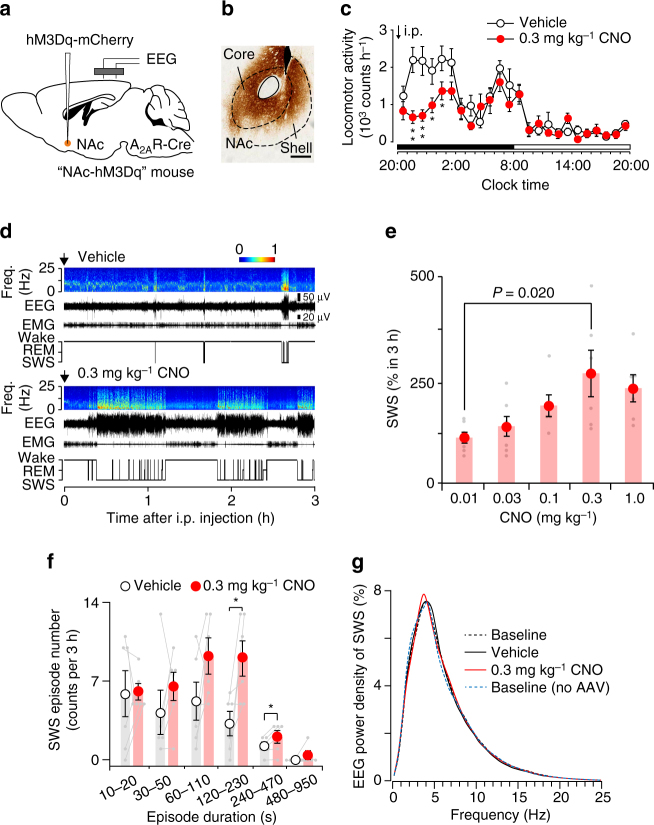
Chemogenetic stimulation of A_2A_R neurons in the NAc induced SWS. **a** A_2A_R-Cre mice were injected with AAV-hSyn-DIO-hM3Dq-mCherry into the NAc and implanted with somnographic electrodes. **b** A brain section was stained against mCherry to confirm that hM3Dq-mCherry protein was expressed in the NAc. Scale bar: 500 μm. **c** Time course of locomotor activity. *Black* and *white bars* indicate dark and light periods, respectively. Two-way repeated measures ANOVA was performed followed by Bonferroni’s post hoc comparisons. *P < 0.05, **P < 0.01, compared with vehicle. **d** Typical examples of EEG (power spectrogram and wave traces), EMG, and hypnograms after the administration of vehicle or CNO. **e** Dose-dependent changes in SWS time normalised to the SWS time of the vehicle control. One-way ANOVA was performed followed by Bonferroni’s post hoc comparisons. **f** SWS episode spectrum. *P < 0.05, compared with vehicle, assessed by paired two-tailed Student’s *t*-test. **g** EEG power density of SWS between 20:00 and 23:00. Data are presented as the mean ± SEM (*n* = 6). Each pair of *grey dots* indicates data from one mouse

We first used a chemogenetic approach to clarify the neurobehavioural and electroencephalographic outcomes of activating A_2A_R-expressing indirect pathway neurons in the NAc. To target excitatory “designer receptors exclusively activated by designer drugs” (DREADD)^19^ in A_2A_R neurons located in the NAc, we used transgenic mice in which Cre-recombinase was expressed under the A_2A_R promoter (A_2A_R-Cre)^20, 21^. Adeno-associated viral vectors (AAV) carrying Cre-recombinase-dependent hM3Dq DREADD (AAV-hSyn-DIO-hM3Dq-mCherry) were stereotaxically injected bilaterally into the NAc of A_2A_R-Cre mice (NAc-hM3Dq mice; Fig. 1a, b). To investigate the effect of NAc A_2A_R neuron stimulation on behavioural activity, we measured locomotor activity after intraperitoneal injections of vehicle or clozapine-N-oxide (CNO), a hM3Dq ligand that evokes neuronal excitation. Locomotor activity was decreased in NAc-hM3Dq mice for 5 h after the administration of CNO at 20:00, i.e., at the beginning of the dark period when mice usually show high levels of arousal (Fig. 1c). We then analysed electroencephalogram (EEG) and electromyogram (EMG) recordings made after vehicle or CNO injections at 20:00 to measure sleep. Compared with vehicle injection, injection of CNO led to a dose-dependent and 3 h-long increase in slow-wave sleep (SWS), which is also known as non-rapid eye movement (non-REM) sleep, the major part of sleep characterised by slow and high-voltage brain waves (Fig. 1d, e and Supplementary Fig. 1a). The number of prolonged SWS episodes (duration between 120–470 s) was also significantly increased (Fig. 1f). Changes in the mean episode number of wakefulness and SWS, and the number of stage transitions from wakefulness to SWS or from SWS to wakefulness did not differ significantly between mice treated with vehicle or CNO for 3 h after the intraperitoneal injection, as assessed by paired Student’s *t*-test (Supplementary Fig. 1b, c). To assess whether EEG activity was altered by chemogenetic activation, we compared the normalised EEG power spectrum of SWS in NAc-hM3Dq mice at baseline (1 day prior to vehicle injection) and after treatment with vehicle or CNO, and in A_2A_R-Cre mice without AAV injections at baseline (Fig. 1g). The EEG activity in the frequency range of 0.5–25 Hz during SWS was indistinguishable between chemogenetically induced and natural (baseline or vehicle treatment) sleep. We also compared the absolute EEG power spectrum of SWS in each condition, but detected no statistical differences (Supplementary Fig. 1d). Moreover, mice treated with vehicle and CNO had similar SWS spindle frequency (Supplementary Fig. 1e). These analyses suggest that the induced sleep was likely physiological sleep rather than abnormal sleep.

### Optogenetic activation of NAc A_2A_R neurons induces SWS

**Fig. 2:**
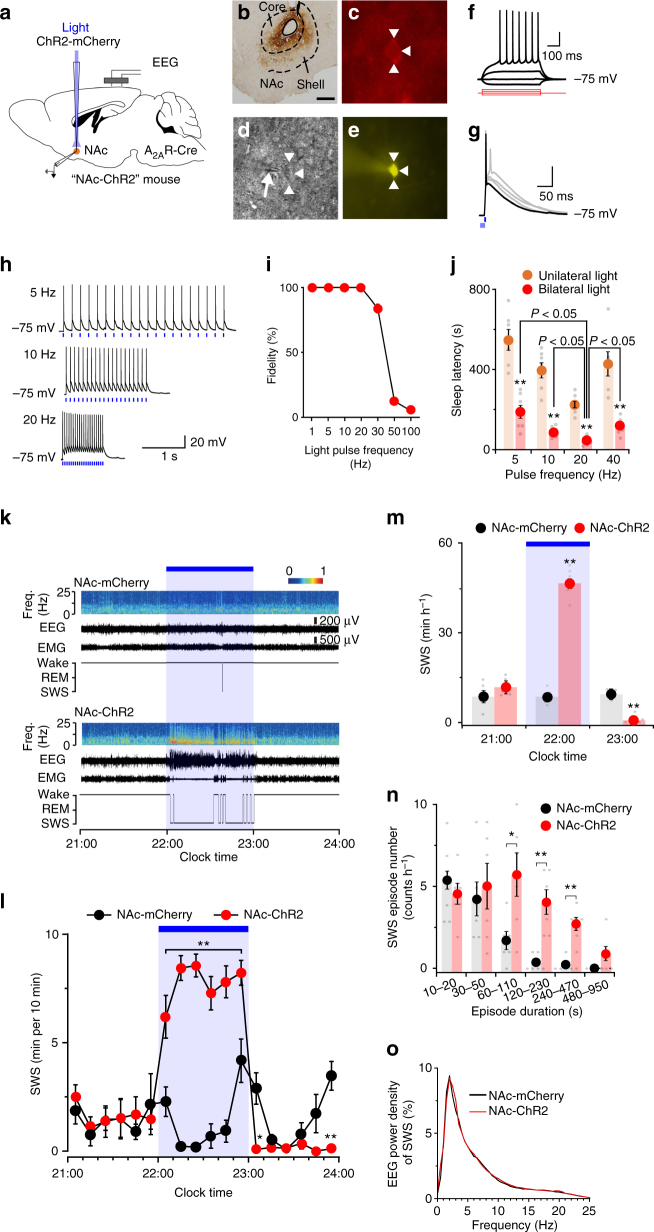
Optogenetic stimulation of A_2A_R neurons in the NAc evoked a rapid and robust SWS response. **a** A_2A_R-Cre mice injected with AAV-EF1α-DIO-ChR2-mCherry into the NAc. **b** A brain section stained against ChR2-mCherry in the NAc. Scale bar: 500 μm. **c**–**e** A typical section of a NAc-ChR2 mouse for patch-clamp electrophysiology showing a ChR2-mCherry-expressing neuron (*white arrowheads*) **c**, the patch pipette (*white arrow*) and the recorded neuron in phase contrast **d**, and the recorded neuron with Lucifer yellow **e**. **f** Recorded neuron showed typical membrane properties and a spiking pattern of medium spiny neurons in response to 500-ms current injections from –40 to 80 pA with 40-pA steps (shown in *red*). **g** Brief 5-ms to 15-ms light pulses at 5-ms intervals evoked single action potentials in ChR2-expressing neurons (shown in *black*). A pulse longer than 15 ms resulted in 2 spikes (shown in *grey*). **h** Responses of an A_2A_R neuron to 20 pulses of *blue light* at various frequencies. Each vertical *blue bar* represents a 5-ms light pulse. **i** Fidelity responses of ChR2-expressing neurons to light pulses at frequencies up to 100 Hz (*n* = 5 cells from 2 mice). **j** Sleep latency after photostimulation in the frequency range of 5–40 Hz. Light was illuminated at least until the mice slept. **P < 0.01 compared between unilateral and bilateral photostimulation, assessed by paired two-tailed Student’s *t*-test. Statistical differences between pulse frequency conditions were assessed by one-way repeated measures ANOVA followed by Bonferroni’s test. **k** Typical examples of EEG (power spectrogram and wave traces), EMG, and hypnograms of two mice injected with AAV-EF1α-DIO-ChR2-mCherry (*lower panel*) or AAV-EF1α-DIO-mCherry (*upper panel*). **l**, **m** The time course **l** and hourly SWS amount **m** during optogenetic experiments. **n**, **o** SWS episode spectrum **n** and EEG power density **o** during photostimulation. Data are presented as the mean ± SEM (*n* = 6). Each *grey dot* indicates data from one mouse. *P < 0.05, **P < 0.01 compared between mouse groups, assessed by unpaired two-tailed Student’s *t*-test **n** or mixed model ANOVA followed by Bonferroni’s post hoc comparisons **l**, **m**. *Blue bars* indicate the period of light illumination

We then used optogenetic stimulation of channelrhodopsin-2 (ChR2), a blue-light-gated cation channel, expressed in NAc A_2A_R neurons to further explore the temporal properties of sleep responses evoked by activating A_2A_R neurons in the NAc. After AAV carrying Cre-dependent ChR2 (AAV-EF1α-DIO-ChR2-mCherry) was stereotaxically injected into the NAc of A_2A_R-Cre mice (NAc-ChR2 mice; Fig. 2a, b), we performed whole-cell patch-clamp recordings of acutely prepared tissue slices containing the NAc (Fig. 2c–i) to test the response of ChR2-expressing neurons, presumably A_2A_R neurons, to optogenetic stimulation. Brief pulses of light (5–15 ms) evoked single action potentials in ChR2-expressing A_2A_R neurons, whereas pulses longer than 15 ms resulted in two spikes (Fig. 2f, g). Light pulses evoked action potentials with high frequency fidelity between 1 Hz and 30 Hz (Fig. 2h, i). We then stimulated the NAc in vivo in NAc-ChR2 mice with 5-ms pulses of blue light in the frequency range of 5–40 Hz at 22:00 when the mice were awake. The latency to sleep onset, defined as the time from the first light pulse to the appearance of the first SWS episode lasting longer than 20 s, was dependent on the pulse frequency as well as on whether the stimulation was unilateral or bilateral, with NAc-ChR2 mice showing the shortest sleep latency when stimulated bilaterally at 20 Hz (Fig. 2j and Supplementary Movies 1, 2). Photostimulation at 20 Hz induced profound SWS, resulting in more than 80% SWS during the illumination period between 22:00 and 23:00 (Figs. 2k–m). After the last light pulse, NAc-ChR2 mice exhibited a significant rebound in wakefulness over the next hour compared with A_2A_R-Cre mice injected with AAV expressing only mCherry (NAc-mCherry mice; Fig. 2l, m). The mice essentially behaved normally during the hour after photostimulation (Supplementary Fig. 2). We used whole-cell patch-clamp recordings in acute brain slices to confirm that optical stimulation of ChR2-expressing A_2A_R neurons at 20 Hz evoked action potentials during a 1-h period (Supplementary Fig. 3). The optogenetically induced firing was sustained at 20 Hz during the initial 5−15 min, depending on the individual neuron, and then progressively decreased to 3.0 ± 1.8 Hz after 1-h stimulation.

A strong shift to prolonged SWS episodes with a duration of 60 s or more was observed in NAc-ChR2 mice (Fig. 2n), resulting in a large increase in the mean duration of SWS episodes (Supplementary Fig. 4a). Moreover, optogenetic stimulation of A_2A_R neurons in the NAc significantly increased the mean number of SWS episodes and the number of wake-to-SWS or SWS-to-wake transitions (Supplementary Fig. 4b, c). The EEG activity in the frequency range of 0.5–25 Hz during SWS episodes was indistinguishable between NAc-ChR2 and NAc-mCherry mice with photostimulation at 20 Hz (Fig. 2o and Supplementary Fig. 4d) as well as between NAc-ChR2 mice with no, 5-Hz, or 10-Hz photostimulation (Supplementary Fig. 4e). Moreover, the number of SWS spindles during photostimulation was similar between NAc-ChR2 and NAc-mCherry mice (Supplementary Fig. 4f). These data suggest that optogenetic activation of A_2A_R neurons in the NAc induced physiological sleep rather than abnormal sleep.

### Activation of NAc core A_2A_R neurons induces SWS

**Fig. 3:**
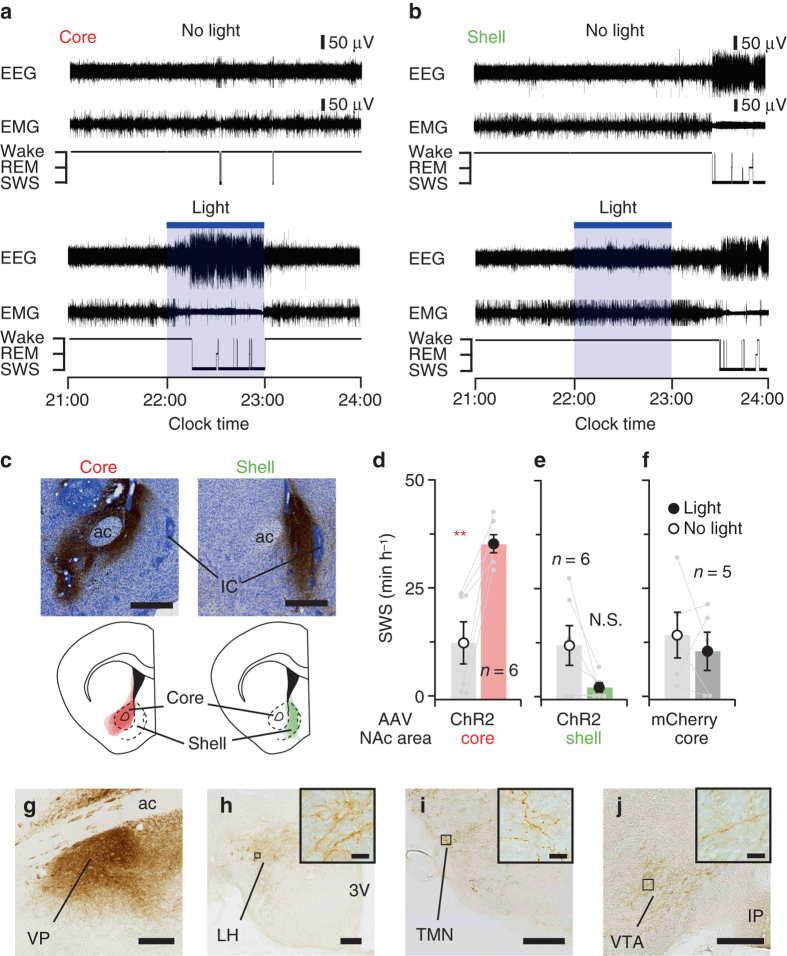
Optogenetic stimulation of A_2A_R neurons in the NAc core, but not the shell, produced SWS. **a**, **b** Typical examples of EEG, EMG and hypnograms of two mice injected with AAV-EF1α-DIO-ChR2-mCherry in the NAc core **a** or shell **b**. Photostimulation of the NAc core, but not the shell, induced SWS (*bottom panels*), as compared to baseline sleep of the two mice without photostimulation (*top panels*). **c** Typical sections from mice with AAV injections in the core (*left panel*) or shell (*right panel*) were stained for mCherry and Nissl. Scale bars: 500 μm. Drawings of superimposed AAV injection sites in the core (in *red*, *n* = 6) and shell (in *green*, *n* = 6) are shown below the photomicrographs. **d**–**f** Hourly amount of SWS during optogenetic stimulation. Data are presented as the mean ± SEM. Each pair of *grey dots* indicates data from one mouse. **P < 0.01 compared to baseline sleep without photostimulation, assessed by paired two-tailed Student’s *t*-test. **g**–**j** Brain sections of a mouse expressing ChR2 in the NAc core cell bodies stained with an antibody against mCherry show ChR2-mCherry positive nerve terminals in the VP **g**, LH **h**, TMN **i**, and VTA **j**. High-magnification photomicrographs of the black rectangular area are shown as insets in **h**–**j**. Scale bars: **g**–**j**, 200 μm; insets in **h**–**j**, 10 μm. Other abbreviations: ac, anterior commissure; IC, islands of Calleja; 3 V, third ventricle; IP, interpeduncular nucleus. N.S., not significant

The two components of the NAc, the core and the shell, have anatomically and functionally distinct projections to the forebrain, diencephalon and brainstem^22–24^. We therefore examined whether sleep is differentially affected by specific stimulation of ChR2-expressing neurons in the core or shell. We unilaterally injected AAV-EF1α-DIO-ChR2-mCherry into the NAc core or shell of A_2A_R-Cre mice and examined the effect of photostimulation on SWS between 22:00 and 23:00. Typical examples of EEG, EMG and hypnograms from mice in the ‘no light’ or ‘light’ conditions are shown in Fig. 3a, b. Photostimulation of ChR2 in A_2A_R neurons in the NAc core strongly increased SWS by 2.9-fold, as compared to the baseline amount of sleep in mice without light stimulation between 22:00 and 23:00 (Fig. 3a, c, d), while SWS was not changed by photostimulation of neurons in the NAc core that expressed only mCherry (Fig. 3f). In contrast, SWS tended to be reduced when only A_2A_R neurons of the medial portion of the NAc shell were stimulated (Fig. 3c, e), although there was no statistically significant difference (*P* = 0.065, paired Student’s *t*-test, *n* = 6).

**Fig. 4:**
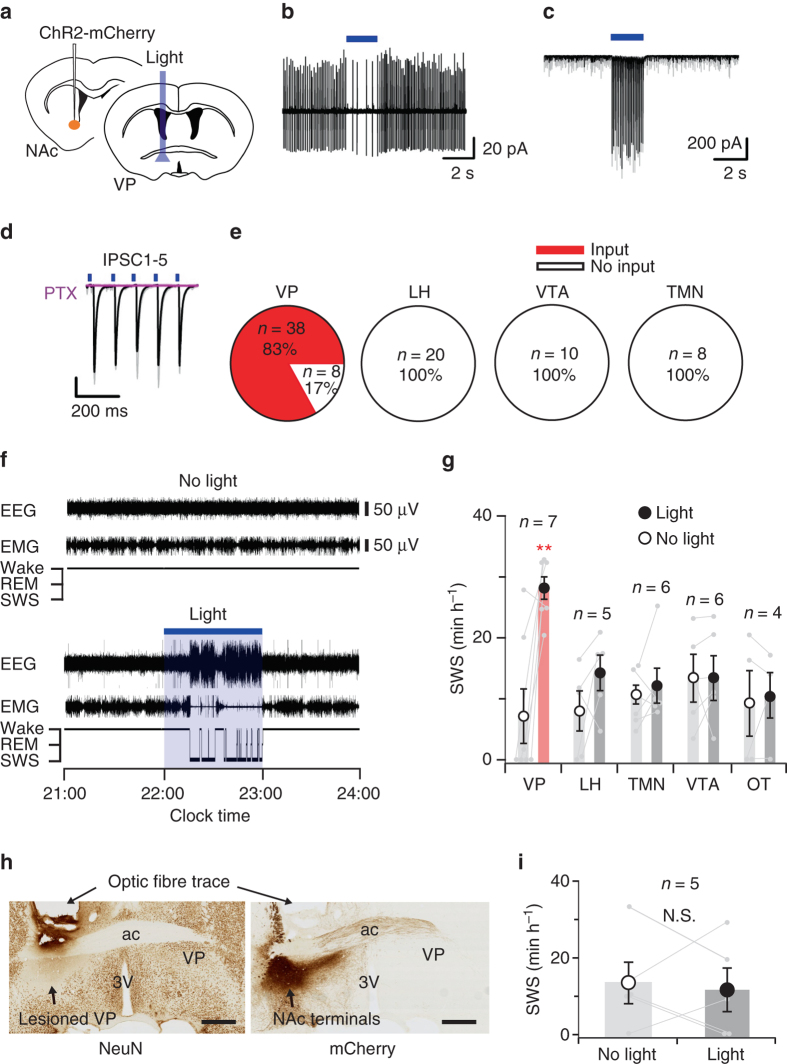
Optogenetic stimulation of ChR2-expressing NAc core axonal terminals in the VP evoked a SWS response. **a** A_2A_R-Cre mice were injected with AAV-EF1α-DIO-ChR2-mCherry into the NAc core and photostimulation occurred at the target side, e.g., the VP. **b** Typical cell-attached recording of a VP neuron in a NAc-ChR2 mouse. Brief light pulses decreased the firing rate. **c** Optical stimulations elicited IPSC in a VP neuron (measured in voltage-clamp at –70 mV). The individual trace is shown in *grey*, whereas the averaged trace is shown in *black*. **d** Optogenetically evoked IPSC were completely blocked in the presence of picrotoxin (PTX: 100 μM; shown in *pink*). **e** Number and proportion of neurons in the VP, LH, VTA and TMN that responded to optical stimulation of ChR2-expressing NAc terminals. **f** Typical examples of EEG, EMG and hypnograms of a NAc-ChR2 mouse, in which photostimulation of ChR2-expressing NAc terminals in the VP induced SWS (*bottom panel*), as compared to baseline sleep in a mouse without photostimulation (*top panel*). **g** Hourly amount of SWS during optogenetic stimulation. Only photostimulation of terminals in the VP induced SWS in NAc-ChR2 mice. **h** Sequential brain sections of a VP-lesioned NAc-ChR2 mouse stained with neuronal marker NeuN (*left panel*) and mCherry (*right panel*) show neuronal cell loss in the VP portion, while this area is innervated by ChR2/mCherry-expressing NAc core terminals. Scale bars: 500 μm. **i** Hourly amount of SWS during VP photostimulation in VP-lesioned NAc-ChR2 mice. Data are presented as the mean ± SEM. Each pair of *grey dots* indicates data from one mouse. **P < 0.01 compared to baseline sleep of NAc-ChR2 mice without photostimulation, assessed by paired two-tailed Student’s *t*-test (**g**, **i**). *Blue bars* indicate the period of light illumination. Other abbreviation: OT, olfactory tubercle; ac, anterior commissure; 3 V, third ventricle. N.S., not significant

NAc A_2A_R neurons are thought to release the neurotransmitter γ-aminobutyric-acid (GABA) upon activation and may thus induce sleep by inhibiting postsynaptic targets. Brain sections of A_2A_R-Cre mice injected with AAV-EF1α-DIO-ChR2-mCherry into the NAc core that were stained with an antibody against mCherry revealed that ChR2-mCherry-expressing core neurons heavily innervated the VP (Fig. 3g). These neurons also made sparse to moderate projections to well-known arousal-promoting areas, such as the lateral hypothalamus (LH), which produces orexin; the tuberomammillary nucleus (TMN), which produces histamine; and the ventral tegmental area (VTA), which produces dopamine (Fig. 3h–j). We therefore determined the extent to which the VP, LH, TMN and VTA contribute to the sleep control mediated by the NAc core. We first performed whole-cell patch-clamp experiments in acute slices to test the responses to optogenetic stimulation of ChR2-expressing NAc core terminals in the VP (Fig. 4a–e). Stimulation (5-ms pulses, 10 Hz) of the NAc core terminals decreased the firing rate and evoked inhibitory postsynaptic currents (IPSC) in most of the recorded VP neurons (Fig. 4b–e and Supplementary Fig. 5a–d), while no such responses were observed in the LH, TMN or VTA (Fig. 4e), possibly due to the low density of synaptic connections on hypothalamic (i.e., LH and TMN) and midbrain VTA neurons. These findings suggest prominent functional synaptic connectivity between the ChR2-expressing NAc core and VP neurons.

Next, we examined the effects of photostimulation of ChR2-expressing NAc core terminals in the VP, LH, TMN or VTA on sleep/wake behaviour in A_2A_R-Cre mice. Light stimulation for 1 h in the VP portion containing ChR2-expressing NAc core terminals remarkably increased SWS by 4.0-fold (Fig. 4f, g), an effect that was similar to the SWS increase evoked by photostimulation of cell bodies in the NAc core (Fig. 3d), although stimulation of ChR2-expressing NAc core terminals in the LH, TMN or VTA did not induce significant changes in SWS (Fig. 4g). IPSC recordings of VP neurons in acute brain slices during 1-h photostimulation revealed that the IPSC frequency was 4.8-fold higher than the baseline at the onset of photostimulation, but returned to the baseline level after 40–50 min (Supplementary Fig. 5e–g). Because the distance between the AAV injection site in the NAc and photostimulation site in the VP is only 0.8 mm, it is conceivable that the changes in SWS resulted from photostimulation of ChR2 in the NAc core cell bodies rather than their axonal terminals in the VP. Hence, we also stimulated an area in the olfactory tubercle 0.8 mm anterior to the NAc core, which served as an anatomical control site. The amount of SWS sleep, however, remained unchanged (*P* > 0.05, Wilcoxon signed-rank test, *n* = 4; Fig. 4g). To exclude the possibility that VP terminal stimulation increased SWS by antidromic activation of the NAc core cell bodies, we unilaterally lesioned VP cells using the neurotoxin ibotenic acid in mice expressing ChR2 in the ipsilateral NAc core (Fig. 4h, left panel). Photostimulation of ChR2-expressing NAc terminals in the VP of those mice (Fig. 4h, right panel) did not affect SWS (Fig. 4i), indicating that the VP is necessary for inducing SWS. Optical stimulation of ChR2 in NAc A_2A_R neurons also evoked IPSC in NAc neurons that did not express ChR2 (Supplementary Fig. 6a–d). This observation may indicate collateral innervation between NAc neurons^25^, which likely include VP-projecting GABAergic neurons of the direct pathway^26^. GABAergic neurons are the main neuronal population in the VP^27^ and therefore, we tested whether disinhibition of these neurons induces SWS. We chemogenetically activated VP GABAergic neurons using AAV-mediated expression of DREADD hM3Dq in mice expressing Cre-recombinase under the promoter of the glutamate decarboxylase 2 (Gad2) gene which is expressed in neurons that use GABA as a neurotransmitter, to catalyse the decarboxylation of glutamate to GABA (Supplementary Fig. 6e, f). The total amount of SWS, however, was suppressed for 3 h after administering 1 mg kg^−1^ CNO to VP-hM3Dq mice, suggesting that SWS is not likely to be induced after disinhibition of VP neurons, for example, by inhibiting VP-projecting GABAergic NAc neurons.

### NAc core A_2A_R neurons are necessary for SWS

**Fig. 5:**
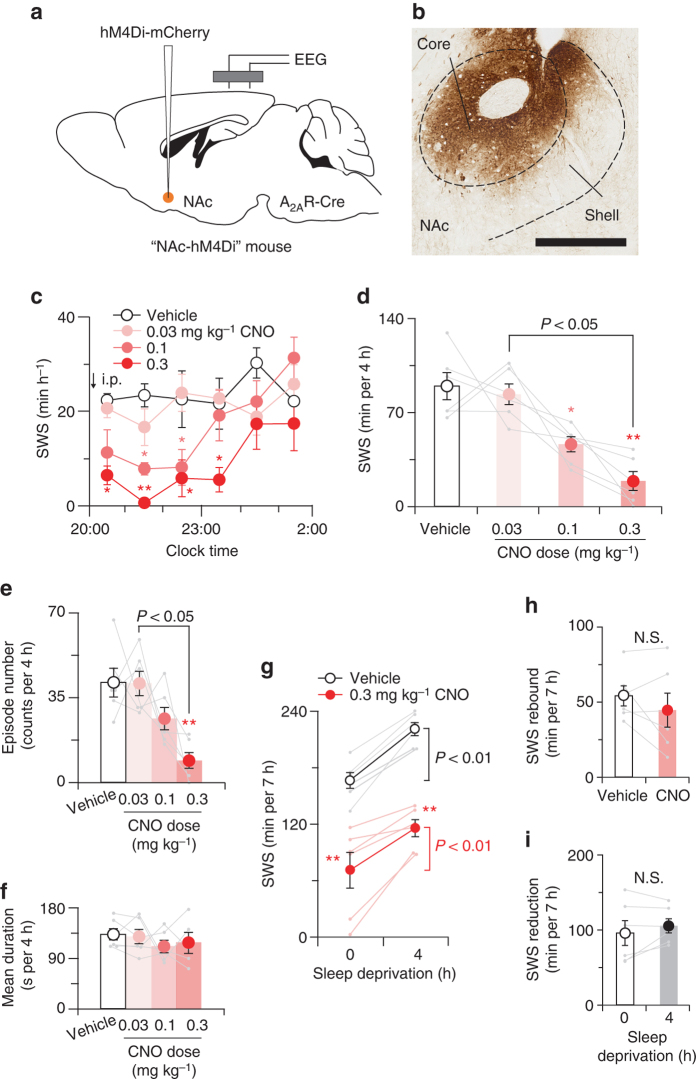
Chemogenetic inhibition of A_2A_R neurons in the NAc core reduced sleep. **a** A_2A_R-Cre mice were injected with AAV-hSyn-DIO-hM4Di-mCherry into the NAc core. **b** Brain sections were stained against mCherry to confirm that hM4Di-mCherry protein was expressed in the NAc core. Scale bar: 500 μm. **c** Time course of SWS. **d** CNO decreased dose-dependently the total amount of SWS. **e**, **f** CNO decreased number of SWS episodes **e** without affecting the mean duration **f**. **g** SWS amount for 7 h after sleep deprivation in the absence and presence of CNO. **h**, **i** SWS rebound after sleep deprivation **h** and CNO-induced SWS reduction **i** were not affected by CNO administration and sleep deprivation, respectively. Data are presented as the mean ± SEM (*n* = 6). Each pair of *grey dots* indicates data from one mouse. *P < 0.05, **P < 0.01 compared with vehicle, assessed by two-way **c**, **g**, one-way **d**–**f** repeated measures ANOVA followed by Bonferroni’s post hoc comparisons, or paired two-tailed Student’s *t*-test **h**, **i**. N.S., not significant

To examine the necessity of NAc core A_2A_R neurons for physiological sleep/wake behaviour, we chemogenetically inhibited these neurons using AAV-mediated expression of inhibitory DREADD hM4Di, which suppresses neuronal activity when CNO is applied^19^, in the NAc core of A_2A_R-Cre mice (NAc-hM4Di mice; Fig. 5a, b). We intraperitoneally administered CNO in NAc-hM4Di mice at 20:00, and CNO dose-dependently reduced SWS for up to 4 h (Fig. 5c, d). The total amount of SWS was suppressed by 80% for 4 h with the highest dose of CNO (i.e., 0.3 mg kg^−1^) compared to vehicle treatment (Fig. 5d). The number of SWS episodes was decreased for 4 h after CNO administration (Fig. 5e), whereas the mean duration of SWS was not changed (Fig. 5f). These results suggest that NAc core A_2A_R neuron activity is necessary to induce SWS under baseline conditions. Moreover, we investigated animal behaviours for over 1 h after CNO administration (Supplementary Fig. 7) and found that CNO significantly increased foraging behaviour (including digging and sniffing) compared to vehicle treatment. CNO administration tended to decrease other behaviours, such as grooming and eating, but the difference did not reach statistical significance (*P* = 0.084 for grooming, *P* = 0.35 for eating, mixed model analysis of variance (ANOVA) followed by Bonferroni’s test, *n* = 6).

We also examined the role of NAc core A_2A_R neurons for the homoeostatic sleep rebound after sleep deprivation by administering vehicle or CNO to NAc-hM4Di mice kept awake for 4 h prior to the drug treatment at 20:00. We observed that vehicle- and CNO-treated mice had a significant SWS rebound during the 7 h after sleep deprivation and that the CNO-induced decrease of SWS was similar in sleep-deprived and non-sleep-deprived animals during the same period (Fig. 5g). Two-way repeated measures ANOVA revealed no statistically significant interaction between sleep deprivation and CNO treatment (*F*_(1,5)_ = 1.53, *P* = 0.272, *n* = 6). Moreover, the amount of SWS rebound after sleep deprivation or CNO-induced SWS reduction during 7 h was not affected by CNO treatment or sleep deprivation, respectively (Fig. 5h, i). These results suggest that the ability of NAc core A_2A_R neurons to induce sleep is independent of homoeostatic sleep pressure.

### Motivational stimuli suppress NAc neuron activity

**Fig. 6:**
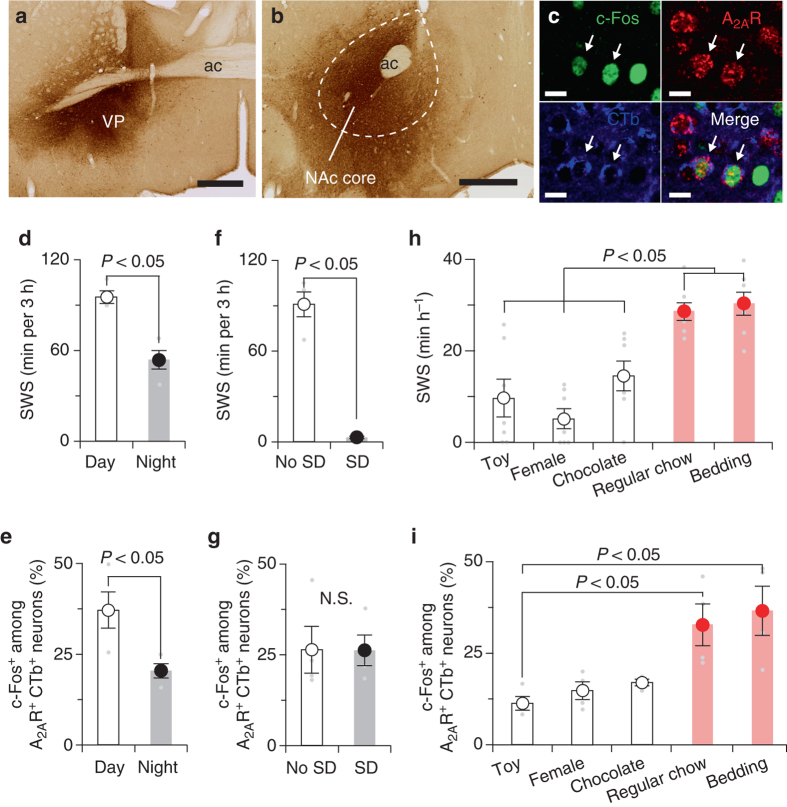
Motivational stimuli suppressed SWS and c-Fos expression in NAc core A_2A_R neurons projecting to the VP. **a** Injection site of retrograde tracer CTb in the VP. **b** CTb-labelled cells in the NAc core. **c** Example of triple-labelled cells (indicated by *arrows*) after immunostaining for c-Fos (*green*) and CTb (*blue*) and in situ hybridisation for A_2A_R (*red*). **d**, **f** SWS during 3 h prior to killing of mice with undisturbed sleep−wake behaviour **d** or deprived of sleep for 4 h **f**. **e** NAc core A_2A_R neurons projecting to the VP exhibited higher c-Fos expression during daytime. **g** 4-h sleep deprivation (SD) did not affect c-Fos expression in NAc core A_2A_R neurons projecting to the VP. **h**, **i** SWS **h** and c-Fos expression in NAc core A_2A_R neurons projecting to the VP **i** in the presence of motivational stimuli (toy, female or chocolate) and non-motivational stimuli (regular chow or bedding). Data are presented as the mean ± SEM (*n* = 7 for **h**; *n* = 4 for the other). Statistical differences were assessed using the Mann−Whitney test **d**–**g**, one-way ANOVA followed by Bonferroni’s test **h**, or Kruskal−Wallis test followed by Dunn’s test **i**. N.S., not significant. Scale bars: **a** and **b**, 500 μm; **c**, 10 μm

Finally, we investigated the patterns of spontaneous activity of the indirect NAc core-VP pathway under various conditions of sleep and motivation using immunohistochemical examination of the expression of c-Fos, a marker of neuronal activation, in mice injected with the retrograde tracer cholera toxin subunit b (CTb) into the VP (Fig. 6a, b). Mice that were killed during the light phase at 12:00 spent more time (95 ± 4.1 min) asleep during the 3 h prior to tissue collection than mice that were killed during the dark phase at 24:00 when they slept 54 ± 6.2 min during the 3 h prior to tissue collection (Fig. 6d). Triple labelling for c-Fos, A_2A_R, and CTb (Fig. 6c) revealed that mice killed during the day had a markedly increased number of VP-projecting NAc core A_2A_R neurons expressing c-Fos (37 ± 5.0%) compared to mice that were killed during the night with c-Fos expression in 20 ± 2.0% of VP-projecting NAc core A_2A_R neurons (Fig. 6e). To determine the role of homoeostatic sleep factors on the activity of the indirect NAc core-VP pathway, we deprived mice of sleep for 4 h before the dark period (Fig. 6f). The c-Fos expression in VP-projecting NAc core A_2A_R neurons of sleep-deprived mice was not different from that in non-sleep-deprived mice (Fig. 6g), however, suggesting that homoeostatic sleep pressure does not induce the activity of VP-projecting NAc core A_2A_R neurons. This observation is consistent with our finding that chemogenetic inhibition NAc core A_2A_R neurons did not affect sleep rebound after sleep deprivation (Fig. 5g–i). Because the NAc is a critical brain area for motivational behaviour^17, 18^, we also examined SWS amount and activity of VP-projecting NAc core A_2A_R neurons in the presence of motivational stimuli (e.g., toy, female mouse or chocolate) or stimuli that are unlikely associated with motivational behaviour (e.g., regular chow or bedding). The amount of SWS in mice was significantly lower in the presence of motivational stimuli compared to mice with non-motivational stimuli (Fig. 6h). Moreover, the number of NAc core neurons triple-labelled for c-Fos, A_2A_R and CTb was lower in male mice that spent time with a toy, a female mouse, or chocolate compared to mice in the presence of non-motivational stimuli (Fig. 6i). These results suggest that motivational stimuli suppress the activity of VP-projecting NAc core A_2A_R neurons and SWS.

## Discussion

Although the VP is widely considered to be a component of the basal ganglia, it occupies an area historically known as the sub-commissural substantia innominata of the basal forebrain^28^. The basal forebrain contains intermingled GABAergic, cholinergic and glutamatergic cell groups, with GABAergic neurons comprising the largest population of nerve cells. Recent findings revealed a major contribution of GABAergic neurons to wakefulness^29^. Using chemogenetic experiments, Anaclet et al.^29^ demonstrated that activating GABAergic neurons in the basal forebrain, which includes the VP, produces wakefulness, whereas inhibition increases sleep. Therefore, it appears that the NAc core produces sleep by inhibiting VP GABAergic neurons. The circuitry by which basal forebrain GABAergic neurons, including the VP neurons, regulate sleep and wakefulness remains largely unresolved. A plausible explanation is that basal forebrain GABAergic neurons inhibit cortical GABAergic interneurons or deep layer pyramidal cells^30, 31^ and thus, induce wakefulness by disinhibiting the cerebral cortex.

In addition to the excitatory afferents to neurons of the indirect pathway in the NAc, adenosine is an obvious candidate for activating NAc core neurons because sufficient levels of adenosine, a well-described endogenous somnogen^15^, are available under basal conditions and excitatory A_2A_R are abundantly expressed in the NAc^32, 33^. The tonic sleep drive by neurons in the NAc core may be inhibited by ongoing cognitive and emotional stimuli, but in the absence of such stimuli may allow the brain to fall asleep by further depressing arousal circuit activity in the basal forebrain. The reduced activity in the arousal systems would then release the ventrolateral preoptic sleep-promoting neurons from inhibition, further promoting sleep. This interpretation is consistent with previous work^13^ showing that injections of an A_2A_R agonist into the preoptic region induces sleep accompanied by c-Fos expression in both ventral striatal neurons and the ventrolateral preoptic nucleus, despite the lack of A_2A_R in the ventrolateral preoptic nucleus.

Caffeine, the most widely consumed wake-promoting psychostimulant in the world, is a nonselective adenosine receptor antagonist^34^ and at least in rats, mainly produces its arousal effect through A_2A_R in the NAc shell^14, 35^. Our data, however, indicate that optogenetic activation of the NAc shell does not induce SWS. As there are functional differences between the NAc shell and core in mediating reward behaviour^22, 36, 37^, it is possible that the two structures also have different functions for sleep–wake regulation. Caffeine appears to produce behavioural activity that induces wakefulness by blocking adenosine at A_2A_R on NAc shell neurons, while activation of NAc core neurons can induce sleep.

The neuronal and molecular bases of the need for sleep or, alternatively, the homoeostatic sleep drive remain unresolved. One potential mechanism is that the gradual accumulation of one or more endogenous somnogenic factors, including prostaglandins, cytokines and adenosine, during wake underlies the propensity for homoeostatic sleep^38^. We demonstrated that the homoeostatic rebound response to restricted sleep is not regulated by NAc A_2A_R neurons and thus other brain regions appear to mediate the homoeostatic sleep pressure.

On the other hand, NAc A_2A_R neurons also express inhibitory dopamine D_2_ receptors (D_2_R). Because VTA dopamine plays a key role in reward and motivational behaviours^17, 18^, it is possible that dopamine release from VTA neurons is the reason that motivational stimuli inhibit NAc core A_2A_R neurons that project to the VP and thus, suppress SWS by releasing VP cells from inhibition. This notion is consistent with findings from recent papers demonstrating an important role of dopaminergic VTA neurons in promoting wakefulness^39, 40^. Moreover, chemogenetic inhibition of NAc core A_2A_R/D_2_R neurons suppresses SWS while promoting foraging, a typical motivational behaviour. Taken together, these observations may suggest that the NAc is a key area where sleep and behavioural responses to motivational stimuli are reciprocally regulated.

## Methods

### Genetic mouse model

We used two genetic mouse lines on a C57BL/6 background in which Cre-recombinase was expressed under the promoter of the A_2A_R gene (A_2A_R-Cre mouse^20^) or Gad2 gene (Gad2-Cre mouse^41^). For behavioural studies, male mice (8–20 weeks) weighing 25–35 g at the beginning of the experiments were housed in insulated sound-proof recording chambers maintained at an ambient temperature of 23 ± 1°C with a relative humidity of 60 ± 2% on an automatically controlled 12-h light/dark cycle (light on at 8:00), and were provided free access to food and water. The numbers of mice used in each experiment were selected based on the expected variations between animals and variability in AAV microinjections. No method of randomisation or blinding was used in any of the experiments. Mice were housed individually after AAV injections. All experiments were performed in accordance with the Animal Care Committee of the University of Tsukuba and Shanghai Medical College of Fudan University, and the National Institutes of Health Guide for the Care and Use of Laboratory Animals, and every effort was made to minimise the number of animals used, as well as any pain and discomfort.

### Plasmids

The plasmids pAAV-hSyn-DIO-hM3Dq-mCherry^42^, pAAV-hSyn-DIO-hM4Di-mCherry^42^ and pAAV-EF1α-DIO-hChR2(H134R)-mCherry^43^ were kindly provided by Dr. Bryan Roth (University of North Carolina School of Medicine, Chapel Hill, NC, USA) and Dr. Karl Deisseroth (Stanford University, Stanford, CA, USA), respectively. To generate the pAAV-EF1α-DIO-mCherry plasmid, the hChR2(H134R)-mCherry fragment between AscI and NheI restriction sites in pAAV-EF1α-DIO-hChR2(H134R)-mCherry was replaced by a polymerase chain reaction (PCR)-amplified fragment containing the mCherry coding sequence.

### AAV generation

The AAV of serotype rh10 for AAV-hSyn-DIO-hM3Dq-mCherry (AAV-M3-DREADD), AAV-hSyn-DIO-hM4Di-mCherry (AAV-M4-DREADD), AAV-EF1α-DIO-ChR2-mCherry (AAV-ChR2) and AAV-EF1α-DIO-mCherry (AAV-mCherry) were generated by tripartite transfection (AAV-rep2/caprh10 expression plasmid, adenovirus helper plasmid, and pAAV plasmid) into 293A cells. After 3 days, the 293A cells were resuspended in artificial cerebrospinal fluid (aCSF), freeze-thawed four times, and treated with benzonase nuclease (Millipore) to degrade all forms of DNA and RNA. Subsequently, the cell debris was removed by centrifugation and the virus titre in the supernatant was determined using an AAVpro Titration Kit for Real Time PCR (Takara).

### Surgery

Surgeries for brain microinjections were conducted under pentobarbital anaesthesia (60 mg kg^−1^, intraperitoneal (i.p.)). Using aseptic techniques, the mice were injected stereotaxically into the NAc with recombinant AAV-M3-DREADD (250 nl per injection, 1.1 × 10^11^ particles ml^−1^), AAV-M4-DREADD (250 nl per injection, 1.1 × 10^11^ particles ml^−1^), AAV-ChR2 (250 nl per injection, 1.4 × 10^11^ particles ml^−1^) or AAV-mCherry (250 nl per injection, 2.4 × 10^11^ particles ml^−1^) with a glass micropipette and an air pressure injector system^44^. The following coordinates were used for injections into the NAc of A_2A_R-Cre according to the atlas of Paxinos and Franklin^45^. For AAV-ChR2, unilateral or bilateral injections were made at 1.2 mm anterior and 0.7–1.3 mm lateral (core/shell: 1.0 mm; core: 1.3 mm; shell: 0.7 mm) to bregma and 4.0 mm below the dura, and for AAV-M3-DREADD, bilateral injections were made 1.3 mm anterior and 1.0 mm lateral to bregma, and 4.0 mm below the dura. For AAV-M4-DREADD, bilateral injections were made 1.2 mm anterior and 1.3 mm lateral to bregma and 4.0 mm below the dural surface. For VP cell lesions or retrograde NAc labelling, mice were unilaterally injected into the VP (0.4 mm anterior and 1.3 mm lateral to bregma, 4.3 mm below the dura) with 5% ibotenic acid (30 nl per injection; Tocris, 0285) or 0.2% CTb (18 nl per injection; List Biological, Cat# 104), respectively. For chemogenetic activation of the VP, AAV-M3-DREADD was bilaterally injected into the VP (0.4 mm anterior and 1.3 mm lateral to bregma, 4.3 mm below the dura) in Gad2-Cre mice.

For optogenetic NAc cell-body stimulation, a stainless-steel cannula (700 μm diameter) was placed during surgery above the NAc located 1.2 mm anterior and 0.7–1.3 mm lateral (core/shell: 1.0 mm; core: 1.3 mm; shell: 0.7 mm) to bregma, and 3.0 mm below the dura, so that the tip of the optical fibre was projecting 1 mm from the end of the cannula during light stimulation. For NAc terminal stimulation, the stainless-steel cannula was placed in the olfactory tubercle: 2.0 mm anterior and 1.1 mm lateral to bregma, 3.0 mm deep from the dura; VP: 0.4 mm anterior and 1.3 mm lateral to bregma, 3.0 mm deep from the dura; LH: 1.4 mm posterior and 0.9 mm lateral to bregma, 3.8 mm deep from the dura; TMN: 2.3 mm posterior and 1.1 mm lateral to bregma, 4.3 mm deep from the dura; or VTA: 3.2 mm posterior and 0.3 mm lateral to bregma, 3.1 mm deep from the dura. Cannulas were fixed on the skull using dental cement.

Mice were also chronically implanted with EEG and EMG electrodes for polysomnography, as previously described in ref. ^46^. Briefly, the implant comprised two stainless-steel screws (1 mm diameter) serving as EEG electrodes, one of which was placed epidurally over the right frontal cortex (1.0 mm anterior and 1.5 mm lateral to bregma) and the other over the right parietal cortex (1.0 mm anterior and 1.5 mm lateral to lambda). Two insulated Teflon-coated, silver wires (0.2 mm in diameter), which were placed bilaterally into the trapezius muscles, served as EMG electrodes. Both EEG and EMG electrodes were connected to a microconnector, and the whole assembly was then fixed to the skull with self-curing dental acrylic resin.

### Behavioural experiments

After 2−3 weeks for postoperative recovery and transgene expression, the mice were placed in experimental cages for a 4-days habituation/acclimatisation period and connected with recording leads. EEG/EMG signals were recorded at baseline (i.e., sleep/wake behaviour of a freely behaving mouse) and under different treatment conditions (i.e., chemogenetic and optogenetic stimulation) over a period of several days. For experiments with motivational stimuli, male mice had free access to blocks (rectangular or triangular prisms; one side was approximately 4 cm), a female C57BL/6 wild-type mouse (10–15 weeks old), milk chocolate (one Hershey’s Kiss; Hershey), a few pieces of fresh regular chow, or a pinch of new bedding from 10:00 until the animals were killed.

For DREADD experiments, all A_2A_R-Cre and Gad2-Cre mice were injected with vehicle or CNO (Sigma, C0832) at 20:00 (A_2A_R-Cre mice) and 9:00 (Gad2-Cre mice) for 2 consecutive days. On day 1, mice were treated with vehicle (saline, i.p.) and on day 2, mice were treated with CNO (i.p.). For sleep deprivation experiments, mice were kept awake by cage tapping and exposure to novel objects for 4 h prior to the dark period and treated with vehicle or CNO at the end of the sleep deprivation (20:00). Mice were awake for 99.6 ± 0.1% of the time during sleep deprivation. SWS rebound was calculated based on the difference between SWS amount from 20:00 to 3:00 in the same animal with and without sleep deprivation.

For optogenetic stimulation, blue light (475 nm) was generated by a RETRA light engine (Lumencor) and applied through optical fibres (Lucir, 500 μm diameter). A pulse generator (Grass-Natus Neurology Incorporated) was used to control the duration and frequency of the light pulses. Fibre-optic rotary joints (Lucir) were used for unrestricted in vivo illumination. Blue light power intensity at the tip of the plastic fibre was ~15 mW mm^−2^, measured by a power metre.

For the c-Fos experiments, we placed mice in experimental cages for a 2−3-day-habituation period before killing under the following conditions. (1) Mice were killed during daytime at 12:00 or night at 24:00. (2) Mice were killed at 20:00 after 4-h sleep deprivation or undisturbed sleep–wake behaviour. (3) Mice were killed 2 h after introduction of motivational or non-motivational stimuli at 10:00.

### Vigilance state assessment

The EEG/EMG signals were amplified and filtered (EEG: 0.5–64 Hz, EMG: 16–64 Hz), then digitised at a sampling rate of 128 Hz, and recorded using SLEEPSIGN software^47^ (Kissei Comtec). In addition, locomotor activity was recorded with an infrared photocell sensor (Biotex). The vigilance states were scored offline by characterising 10-s epochs into three stages, including awake, SWS and REM sleep, according to standard criteria^46^. As a final step, defined vigilance stages were examined visually, and corrected when necessary. Spindles during SWS were analysed as previously reported^48^. In brief, EEG was bandpass-filtered (10–13 Hz) to visually identify sleep spindles from the raw EEG signals.

### Behavioural analysis

Animal behaviours after photostimulation or CNO injections were analysed using video recordings as described in a previous report^49^. Briefly, behaviours during wakefulness were scored in 4-s epochs as grooming, foraging (including digging and sniffing), rearing, eating, drinking, ambulation or quiet waking, when the behaviour accounted for more than 50% of the epoch.

### Patch-clamp electrophysiology

A_2A_R-Cre mice were injected with AAV-ChR2 and after 21 days, then anaesthetised and perfused transcardially with modified aCSF containing 213 mM sucrose, 2.5 mM KCl, 1.25 mM NaH_2_PO_4_, 26 mM NaHCO_3_, 10 mM glucose, 2 mM Na-pyruvate, 0.4 mM ascorbic acid, 3 mM MgSO_4_ and 0.1 mM CaCl_2_, and saturated with 95% O_2_ and 5% CO_2_. Coronal brain sections (NAc, VP, LH, etc., thickness: 300 µm) were cut on a vibratome (VT 1200 S, Leica) and transferred to normal aCSF containing 126 mM NaCl, 2.5 mM KCl, 1.25 mM NaH_2_PO_4_, 1 mM MgSO_4_, 2 mM CaCl_2_, 26 mM NaHCO_3_, and 25 mM glucose. Slices were incubated at 32 °C for 30 min and subsequently maintained at room temperature for 30 min before recording. Patch electrodes had a resistance of 4–5 MΩ with an internal solution containing 105 mM potassium gluconate, 30 mM KCl, 4 mM ATP-Mg, 10 mM phosphocreatine, 0.3 mM EGTA, 0.3 mM GTP-Na, and 10 mM HEPES (pH 7.3, 295–310 mOsm). Cell-attached and whole-cell current-clamp and voltage-clamp (held at –70 mV) recordings were made at 30–32 °C using a MultiClamp 700B amplifier (Axon Instruments). Signals were filtered at 4 kHz and digitised at 10 kHz with a DigiData 1440 A (Axon Instruments). Data were acquired and analysed with pClamp10.3 software (Axon Instruments). ChR2 was stimulated by 470-nm light (5-ms pulses, 1–100 Hz) delivered via an optical fibre coupled to a laser source (OEM Laser Systems). The end of the optical fibre was placed 300 μm above the recorded cell. When needed, 25 μM d-(−)-2-amino-5-phosphonopentanoic acid (d-APV), 5 μM NBQX, and 100 μM picrotoxin were added into the aCSF to block NMDA, AMPA and GABA_A_ receptors, respectively. Series resistance compensation was not used, and thus cells with series resistance changes over 25% were discarded.

### Histology

Animals were deeply anaesthetised with an overdose of chloral hydrate (500 mg kg^−1^, i.p.) and perfused through the left ventricle of the heart with saline followed by neutral buffered 10% formalin. Brains were removed and placed in 20% sucrose in phosphate-buffered saline (PBS) overnight at 4 °C to reduce freezing artefact. The brains were then frozen on dry ice and sectioned at 30–40 µm on a freezing microtome.

Immunohistochemistry was performed on free-floating sections as described previously^14^. In brief, sections were rinsed in PBS, incubated in 0.3% hydrogen peroxide in PBS for 30 min at room temperature, and then sequentially at room temperature in 3% normal donkey serum and 0.25% Triton X-100 in PBS (PBT) for 1 h and primary antibody diluted in PBT with 0.02% sodium azide overnight. After overnight incubation with rabbit anti-DsRed antibodies (1:10,000; Clontech, Cat# 632496), mouse anti-NeuN antibodies (1:2000; Millipore, Cat# MAB377) or goat anti-CTb antibody (1:15,000; List Biological, Cat# 703), sections were rinsed and incubated for 2 h in biotinylated antibody (Jackson ImmunoResearch) at a dilution of 1:1000. All tissue sections were then treated with avidin-biotin complex (1:1000; Vectastain ABC Elite kit, Vector Labs) for 1 h, and immunoreactive cells were visualised by reaction with 3,3′-diaminobenzidine and 0.01% hydrogen peroxide. Tissue sections mounted on glass slides were scanned with a Hamamatsu NanoZoomer-XR Digital slide scanner (Hamamatsu Photonics), and digital photomicrographs were analysed with Hamamatsu NDPView software v2.4.26.

For in situ hybridisation, we generated a 476-bp riboprobe for A_2A_R mRNA based on sequences from the Allen brain atlas (http://www.brain-map.org/). Briefly, we amplified the probe fragment by PCR (forward, 5′-CACATCATCAACTGCTTCACCT-3′; reverse, 5′-CATGAGGCTGTTCCTACCCTAC-3′ conjugated with T7 promoter) using mouse brain cDNA as template followed by in vitro transcription to make a digoxigenin-labelled antisense probe. Then, sections were incubated with 1 μg ml^−1^ of the A_2A_R probe at 50 °C overnight, washed in saline sodium citrate buffer, incubated with peroxidase conjugated anti-digoxigenin antibody (1:250; Roche) overnight, and reacted with tyramide-conjugated Cy3 (PerkinElmer).

For triple labelling of c-Fos, A_2A_R mRNA and CTb, we first immunostained c-Fos with rabbit anti-c-Fos antibody (1:2000; Millipore, Cat# ABE457), peroxidase conjugated anti-rabbit antibody (1:250; Jackson ImmunoResearch), and tyramide-conjugated fluorescein (PerkinElmer). We then performed A_2A_R in situ hybridisation as described above and finally visualised CTb immunoreactivity with goat anti-CTb antibody (1:15,000), biotinylated anti-goat antibody (1:500; Jackson ImmunoResearch), and Alexa Fluor 647-conjugated streptavidin (1:500; Invitrogen). Images were analysed with a laser confocal microscope LSM730 (Carl Zeiss).

### Statistical analysis

Data are presented as the mean and standard error of the mean (SEM). Statistical comparisons between two groups were performed using the paired or unpaired two-tailed Student’s *t*-test or Wilcoxon signed-rank test. For *t*-tests, we established the normality of each dataset using the Kolmogorov–Smirnov test. Comparisons among multiple parameters were performed by one-way, two-way or mixed model ANOVA followed by Bonferroni’s post hoc comparisons. In all cases, *P*-values less than 0.05 were considered significant.

### Data availability

The authors declare that all data supporting the findings of this study are available within the paper and its supplementary information files, or are available from the corresponding author upon request.

## Electronic supplementary material

Supplementary Information Supplementary figures

Description of Additional Supplementary Files

Supplementary Movie 1

Supplementary Movie 2
