# Supplementary material for: Slow-wave sleep is controlled by a subset of nucleus accumbens core neurons in mice
Source: Nat Commun. 2017 Sep 29;8:734. doi: 10.1038/s41467-017-00781-4 (PMC5622037; doi:10.1038/s41467-017-00781-4)
Supplement: Supplementary file 1 — Supplementary Information Supplementary figures [file 41467_2017_781_MOESM1_ESM.pdf]

Supplementary figures

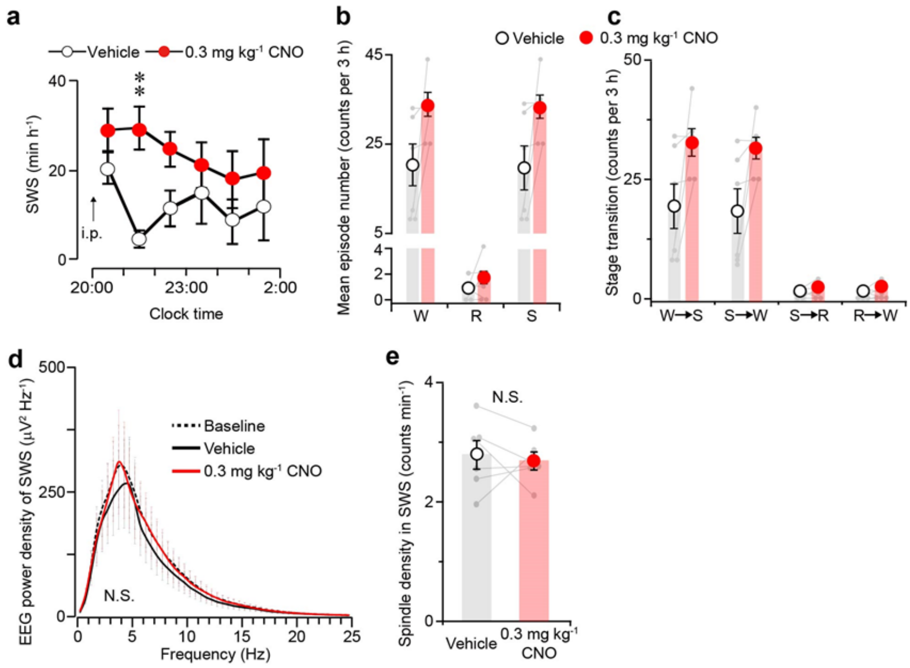

Supplementary Figure 1. **Sleep profile after chemogenetic stimulation of $A_{2A}R$ neurons in NAc-hM3Dq mice.** (**a**) Time-course of SWS; arrow indicates the time of i.p. injection. (**b**, **c**) Effect of DREADD activation of NAc $A_{2A}R$ neurons on mean episode number (**b**) and stage transition (**c**) for 3 h after the administration of vehicle or CNO. (**d**) Absolute EEG power density of SWS between 20:00 and 23:00. (**e**) SWS spindle density. Data are presented as the mean ± SEM (n = 6). Each pair of grey dots indicates data from one mouse. **P < 0.01, compared between vehicle and CNO treatment of NAc-hM3Dq mice, assessed by two-way repeated measures ANOVA followed by Bonferroni's post-hoc comparisons (**a**, **d**) or paired two-tailed Student's t-test (**b**, **c**, **e**). Abbreviations: W, wakefulness; S, SWS; and R, REM sleep. N.S., not significant.

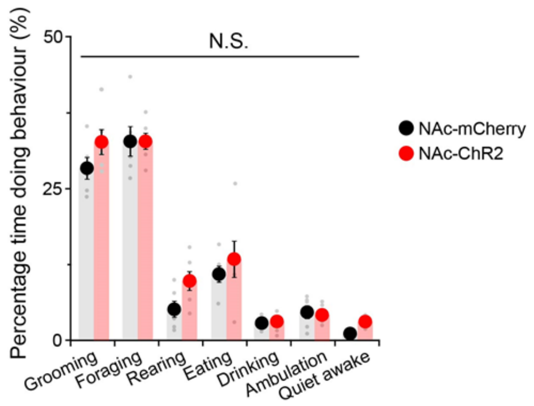

Supplementary Figure 2. Quantification of behaviours observed after photostimulation between 23:00 and 24:00. Data are presented as the mean ± SEM (n = 6). Each grey dot indicates data from one mouse. N.S., not significant, assessed by two-way ANOVA followed by Bonferroni's post-hoc comparisons.

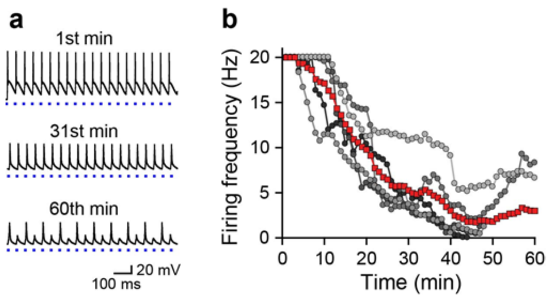

Supplementary Figure 3. Responses of A_{2A}R-neurons to pulses of blue light at 20 Hz during 1-h photostimulation. (**a**) Representative responses of an A_{2A}R-neuron during 1 s in the 1st, 31st, and 60th min of 1-h optical stimulation. Blue bars represent a 5-ms light pulse. (**b**) Time course of action potential frequency in A_{2A}R neurons. Individual neurons are shown in grey, whereas the mean of all neurons is shown in red (n = 5 in 3 mice).

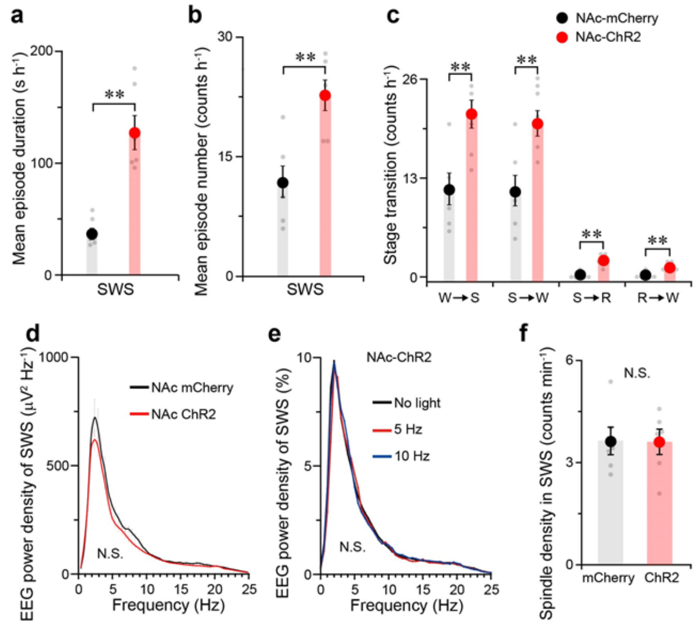

Supplementary Figure 4. Effect of optogenetic stimulation of A_{2A}R neurons in NAc-ChR2 and NAc-mCherry mice on sleep profiles. (**a**–**c**) Mean episode duration (**a**) and number (**b**) of each behavioural stage and sleep–wake stage transitions (**c**) during 1-h light illumination (5 ms, 20 Hz). (**d**) Absolute EEG power density of SWS during photostimulation. (**e**) EEG power density of SWS episodes in NAc-ChR2 mice with or without photostimulation. (**f**) SWS spindle density during photostimulation. Data are presented as the mean ± SEM (n = 6). Each grey dot indicates data from one mouse. $^{**}P < 0.01$, assessed by unpaired two-tailed Student's t-test. Abbreviations: W, wakefulness; S, SWS; and R, REM sleep. N.S., not significant.

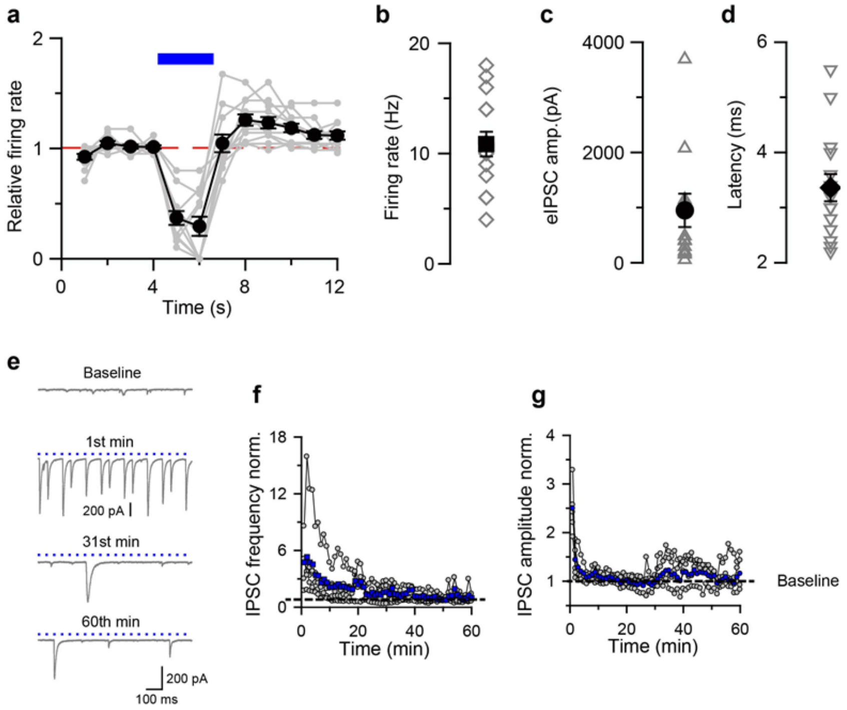

Supplementary Figure 5. Patch-clamp response of VP neurons to optogenetic stimulation of ChR2-expressing NAc terminals. (**a**) Time-courses of the individual (in grey) and mean (in black) firing rates of VP neurons (*n* = 13). Values are normalized by the value of the mean firing rate during the first 4 s. Blue bar: 5-ms blue light pulses at a frequency of 10 Hz. (**b**–**d**) Summary of the individual and mean firing rates of VP neurons (**b**), amplitude of evoked IPSC (**c**) and latency between the first light pulse and the onset of evoked IPSC (**d**). Individual neurons: grey marker; mean of 16 neurons: black marker. (**e**) Representative IPSC traces recorded in a VP neuron (measured in voltage-clamp at –70 mV). IPSC traces during 1 s at baseline and in the 1st, 31st, and 60th min of photostimulation are shown. (**f**, **g**) Time courses of the individual (in grey) and mean (in blue) frequency (**f**) and amplitude (**g**) of IPSC recorded in VP neurons (*n* = 6 in 4 mice). Values are normalized to the baseline (dotted black line).

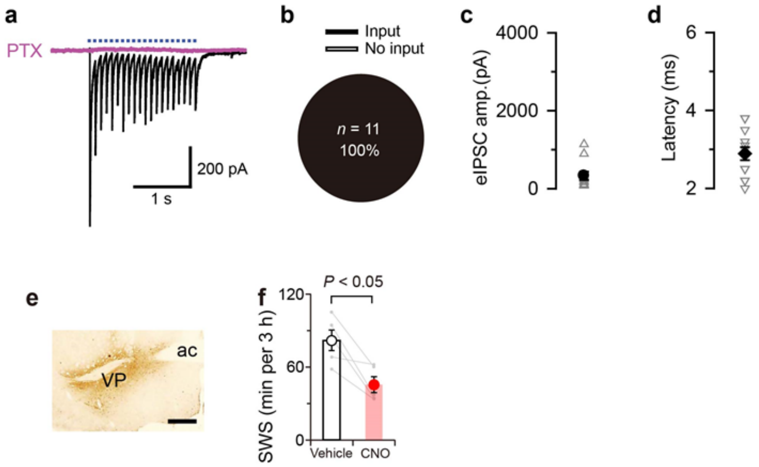

Supplementary Figure 6. **Effect of optogenetic stimulation of NAc $A_{2A}R$ neurons on ChR2-negative NAc neurons.** (**a**) Optogenetic stimulation evoked IPSC in a NAc neuron that did not express ChR2 (measured in voltage-clamp at –70 mV). Optogenetically evoked IPSC were completely blocked in the presence of picrotoxin (PTX: 100 µM; shown in pink). (**b**) Number and proportion of ChR2-negative NAc neurons that responded to optical stimulation of ChR2-expressing NAc $A_{2A}R$ neurons. (**c**, **d**) Summary of the individual and mean amplitudes of evoked IPSC of NAc neurons (**c**) and latency between the first light pulse and the onset of evoked IPSC (**d**). Individual neurons: grey marker; mean: black marker. (**e**, **f**) Chemogenetic activation of VP GABAergic neurons. (**e**) Brain sections were stained with antibodies against mCherry to confirm that hM3Dq-mCherry protein was expressed in the VP of Gad2-Cre mice. Scale bar: 500 µm. ac, anterior commissure. (**f**) 1 mg kg^{-1} CNO decreased SWS amount during 3 h. Data are presented as the mean ± SEM (n = 5). Each grey dot indicates data from one mouse. Significance was assessed by paired two-tailed Student's t-test.

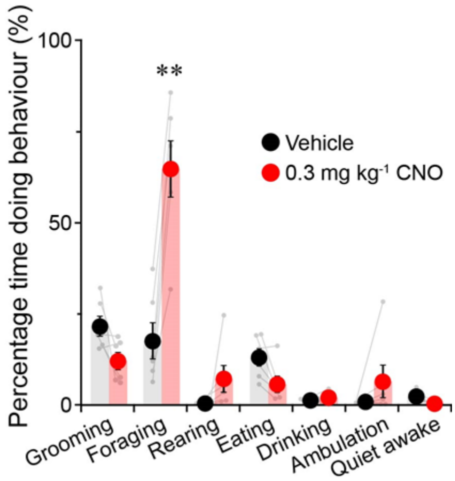

Supplementary Figure 7. Quantification of behaviours observed during chemogenetic inhibition (21:00–22:00). Data are presented as the mean ± SEM (n = 6). Each pair of grey dots indicates data from one mouse. $^{**}P < 0.01$, assessed by mixed model ANOVA followed by Bonferroni's post-hoc comparisons.
