# Supplementary material for: Slow-wave sleep is controlled by a subset of nucleus accumbens core neurons in mice
Source: Nat Commun. 2017 Sep 29;8:734. doi: 10.1038/s41467-017-00781-4 (PMC5622037; doi:10.1038/s41467-017-00781-4)
Supplement: Supplementary file 2 — Description of Additional Supplementary Files [file 41467_2017_781_MOESM2_ESM.pdf]

Description of Additional Supplementary Files

File Name: Supplementary Movie 1
Description: A mouse in which SWS was induced by blue light illumination of NAc neurons expressing ChR2.

File Name: Supplementary Movie 2
Description: A mouse in which SWS was not induced by blue light illumination of NAc neurons expressing only mCherry.
